# Energy‐Based Skin Rejuvenation: A Review of Mechanisms and Thermal Effects

**DOI:** 10.1111/jocd.16657

**Published:** 2024-11-01

**Authors:** Ximeng Jia, Yongqiang Feng

**Affiliations:** ^1^ Laser Aesthetic Center, Plastic Surgery Hospital Chinese Academy of Medical Sciences and Peking Union Medical College Beijing China

**Keywords:** collagen, electromagnetic, energy‐based devices, photoelectric and ultrasonic, skin aging, skin resurfacing and rejuvenation, thermal effect, thermal injury

## Abstract

**Background:**

Energy‐based photoelectric and ultrasonic devices are essential for skin rejuvenation and resurfacing in the field of plastic surgery and dermatology. Both functionality and appearance are impacted by factors that cause skin to age, and various energy types have variable skin penetration depths and modes of transmission.

**Aim:**

The objective is to advise safe and efficient antiaging treatment while precisely and sensitively controlling and assessing the extent of thermal damage to tissues caused by different kinds of energy‐based devices.

**Methods:**

A literature search was conducted on PubMed to review the mechanisms of action and thermal effects of photoelectric and ultrasonic devices in skin remodeling applications.

**Results:**

This paper reviews the thermal effects of energy‐based devices in skin resurfacing applications, including the tissue level and molecular biochemical level. It seeks to summarize the distribution form, depth of action, and influencing factors of thermal effects in combination with the mechanisms of action of various types of devices.

**Conclusion:**

Accurate control of thermal damage is crucial for safe and effective skin remodeling treatments. Thorough investigation of molecular biochemical indicators and signaling pathways is needed for real‐time monitoring and prevention of severe thermal injury. Ongoing research and technological advancements will improve the accuracy and control of thermal damage during treatments.

## 1 Introduction

Skin aging is a complex process involving a combination of stages and factors. This process can affect the structure and function of the skin in the form of wrinkles, sagging, enlarged pores, and abnormal pigmentation. It can be divided into two main types: intrinsic and extrinsic aging. During this process, alterations are observed in both the epidermis and the dermis, with more pronounced differences between the two types of aging in the dermis. Intrinsic aging slowly causes tissue degeneration with age and is also influenced by genes, hormonal changes, and cellular metabolism, resulting in a gradual loss of skin structure and function [1]. This is characterized by a decrease in eosinophilic and Hales staining substances and smaller fibroblasts in the dermis. Extrinsic aging stems mainly from skin damage caused by ultraviolet radiation, known as photoaging. This process leads to the production of reactive oxygen species and genetic alterations. Reactive oxygen species cause an increase in the oxidative stress and lead to a reduction and breakdown of elastic fibers and collagen, which in turn triggers undesirable changes in skin color and texture [2, 3]. Histologically, we can observe large, eosinophilic‐filled areas under the photodamaged dermis [4]. It is crucial to note that chronological aging and photoaging are interconnected processes, jointly contributing to the manifestations of skin aging. Aging makes the skin more vulnerable to photodamage, while UV radiation accelerates the intrinsic aging process.

With the improvement of aesthetic standards, there is a growing demand for skin rejuvenation, particularly in the realm of light, ultrasound, and electricity—energy‐based systems that have given rise to a variety of new technologies and devices. Photoelectric and ultrasonic technology plays a crucial role in plastic surgery and dermatology. In recent years, it has achieved remarkable results in improving skin aging, scarring, acne, pigmentary diseases, skin vascular diseases, and body contouring [5, 6, 7, 8]. It is widely favored for its fast, effective, simple, and minimally invasive nature. The main principle of photoelectric and ultrasonic technology is based on the thermal effect produced in tissues through the conversion of various energies into heat. Controlling tissue thermal damage effectively to achieve a favorable thermal effect is a crucial consideration in the treatment process. Therefore, conducting an objective and standardized assessment of the thermal effects of photoelectric and ultrasonic devices in skin remodeling, and avoiding the risk of complications associated with inappropriate thermal damage, is essential to ensure safe and effective treatment.

## 2 Thermal Effects and Thermal Damage

Skin rejuvenation is a crucial application area for energy‐based devices that thermally stimulate tissues or cells by converting various energies into heat. Ideal thermal stimulation should accurately control the thermal effect on the dermis and subcutaneous fibrous septum or fascia to trigger a series of positive physiological responses beneficial to rejuvenation. These include the activation of fibroblasts, remodeling of collagenous tissue, and revascularization. The magnitude of the tissue temperature increase and the duration of the effect are the two main elements determining the thermal effect on the tissue. When a tissue is exposed to excessive temperature or prolonged heat, the extent of tissue damage exceeds its tolerance range, leading to temporary or permanent abnormal changes in tissue and cell structure, function, and metabolism. These abnormal changes are termed reversible and irreversible thermal injuries, depending on the severity and duration of the thermal stimulus. In clinical practice, the use of photoelectric and ultrasonic devices necessitates precise control of the degree of thermal damage and the induction of controllable thermal damage to elicit beneficial tissue and cellular responses [9, 10, 11, 12]. Understanding the mechanism of thermal stimulation, the distribution of thermal effects, and the factors affecting thermal effects is key to guiding safe and effective treatment. In the following, we discuss the control and mechanism of the thermal effect from various aspects.

### 2.1 Thermal Effects on Tissues and Cells

The optimal ambient temperature for the growth of most mammalian cells is 37°C. Local tissue temperatures ranging from 37°C to 42°C induce tissue congestion. However, this process is safe and does not result in significant skin changes or adverse tissue effects. Studies have consistently demonstrated that even under prolonged exposure, the threshold for cellular thermal damage remains at 43°C. Thermal damage to tissues can occur at temperatures above 43°C. However, the extent of damage is not solely determined by temperature but is contingent on the interplay of temperature and exposure time. With increasing temperature and/or duration of action, one or more of the thermal effects of hyperthermia, coagulation, vaporization, carbonization, and ablation can be induced and produce varying degrees of thermal damage to tissues. It may appear to the naked eye as erythema, pallor, darkening, and ablation of the skin. Microscopically, tissue edema, coagulation necrosis, mechanical rupture, and pyrolysis can be observed [11, 13, 14, 15, 16, 17, 18].

The kinetics of several morphological and physiological indicators of thermal injury are exponentially dependent on temperature and linearly dependent on exposure time. The exposure time required to cause irreversible damage at any given temperature is estimable. For every 1°C above the threshold temperature (43°C), the time to cause thermal injury to the same number of cells reduces by 50%. Tissue lethality takes 120 min at 43°C, but only about 1 s at 56°C [19, 20, 21]. Although utilizing the known thermal effects of tissues at corresponding temperatures can help achieve therapeutic requirements, some potential side effects are difficult to predict. These side effects may include blister formation due to irreversible thermal damage, post‐inflammatory hyperpigmentation, hypopigmentation, and scar formation [22, 23, 24, 25, 26, 27].

The dermal matrix of adult skin consists of collagen I (80%–85%) and III (10%–15%), along with glycosaminoglycans and elastin fibers, which are synthesized and secreted by fibroblasts. Collagen I mainly affects the thickness of the skin, whereas collagen III is more involved in the formation of the skin's meshwork [28]. The aging process is characterized by a decrease in collagen fibers.

Fibroblasts respond differently to various temperatures, which is crucial as they are involved in skin remodeling. Heat exposure at 43°C for 10 min leads to reversible, slight cellular thermal damage, but the damage recovers later and cellular function is enhanced [29]. Thermal stimulation at 45°C and 60°C for 2 s [30, 31] resulted in the upregulation of type I and type III procollagen, and cells under the action of 60°C showed a more persistent trend of upregulation with increasing time while the proliferation rate decreased.

Collagen also respond directly when received thermal stimulation. Rejuvenation can also be induced by controlled denaturation or ablation of tissue in the microthermal zone. Controlled collagen contraction and denaturation have been found to be effective in inducing tissue remodeling [11, 32, 33, 34, 35, 36, 37]. In the early stage of thermal stimulation, with increasing temperature, the collagen fiber helix undergoes partial or complete denaturation and collagen shrinkage, known as thermally induced collagen contraction. This tissue‐tightening effect is most pronounced at 63°C [38, 39, 40]. Within milliseconds, the fractional form of treatment produces a zone of micro‐thermal coagulation or vaporization, causing the surrounding cells to synthesize and exude extracellular matrix such as collagen. The thermocoagulated or vaporized zone, as well as the surrounding sub‐thermal zone, produce new collagen, allowing for tissue repair and remodeling [18, 33, 41, 42, 43]. Photoelectric and ultrasonic technology has been found to reverse skin aging by activating cell proliferation and cellular activity, increasing the expression of both collagen I and III [3, 44, 45].

### 2.2 Thermal Effects at the Molecular Biochemical Level

Heat stimulation does not usually produce major tissue changes. Sensitive biological markers are required to better understand the cellular response, sublethal heat injury, and dynamic histological heat response. Heat injury affects cells and tissues through a variety of biological processes and communication pathways. Heat shock proteins (HSPs) are among the most common indicators of thermal injury, and they play an important role in the response. HSPs function by aiding normal protein folding, inhibiting protein aggregation and degradation, and participating in cellular stress response and repair processes. Heat injury causes a considerable increase in HSP expression, particularly for subtypes such as HSP70, HSP47, and HSP27. These HSPs were activated and regulated upon thermal injury to assist cells in dealing with heat stress‐induced protein denaturation and intracellular disarray. For instance, at 42°C, HSP 70 temporarily activates expression; at 43°C and higher, expression increases quickly and significantly. It is the primary heat‐stress‐responsive protein and a significant indication of the degree of protein denaturation in cells. HSP 47 expression increases at temperatures above 42°C, and its level is linked to the rate of collagen synthesis. It also has a greater impact on the process of long‐term wound healing. In response to heat stress (42°C or above), HSP27's mRNA expression increased rapidly, but protein expression did not alter much. Other HSP isoforms, such as HSP 72 and HSP 60, have variable expression patterns as temperatures change. Tissue‐specific HSP72 expression was upregulated at 42°C, and the degree of heat stress influenced the level of expression. However, HSP 60 was less sensitive to temperature variations, with no significant change in expression levels following a 43°C heat shock [22, 31, 46, 47, 48, 49, 50, 51, 52]. Furthermore, HSPs regulate heat stress‐induced inflammatory responses, prevent apoptosis, and modulate intracellular signaling pathways [53].

The thermal effect may impact several important signaling pathways within skin cells. In terms of the inflammatory response, heat injury can trigger the release of inflammatory factors, leading to a significant upregulation of expression levels of inflammatory cytokines such as tumor necrosis factor‐α (TNF‐α), interleukin‐6 (IL‐6), and interleukin‐1β (IL‐1β). These inflammatory factors, through the activation of inflammatory signaling pathways such as NF‐κB and MAPK, participate in regulating the inflammatory response induced by heat stress. These changes play a crucial role in inflammation‐mediated cellular damage and repair [54, 55, 56, 57]. In terms of oxidative stress, heat injury can lead to increased oxidative stress, affecting the intracellular redox balance. The changes in superoxide dismutase, catalase, malondialdehyde, etc. reflect the alteration of the intracellular oxidative stress state. The Nrf2 antioxidant signaling pathway, PI3K‐Akt, and other signaling pathways affect the cell's ability to cope with oxidative stress [58, 59, 60, 61, 62]. In terms of apoptosis, heat injury may lead to increased cell apoptosis. This can be evaluated by detecting the activity of apoptosis‐related genes such as Bcl‐2, Bax, and the Caspase family. These genes are involved in the apoptotic signaling pathways of cells, including the mitochondrial pathway, death receptor pathway, etc., affecting the cell survival and death decisions [63, 64, 65, 66, 67, 68, 69]. Additionally, MAPK, Wnt/β‐catenin, and TGF‐β signaling pathways have an impact on important biological processes such as the development, regeneration, cell proliferation, and differentiation of skin tissue [70, 71, 72, 73, 74, 75].

In conclusion, the changes in these genes and biological indicators reflect the biological effects of heat injury, involving multiple biological processes and signaling pathways such as the inflammatory response, oxidative stress, cell apoptosis, as well as inflammation and immune responses. In‐depth research and analysis of these indicators contribute to a more comprehensive understanding of the biological mechanisms of heat injury, providing important evidence for the assessment, real‐time monitoring, and prevention of excessive heat damage.

## 3 Distribution and Depth of Thermal Effect

Changes in the different facial tissue are strongly linked to the various signs of aging. The primary causes of skin aging symptoms include decreased epidermal cell metabolic activity, aging and loss of collagen and elastic fibers in the dermis, abnormal microvascular function, and deeper changes like subcutaneous fat volume loss and weakening of the superficial musculoaponeurotic system (SMAS). Generally, the thickness of facial skin is 2–3 mm, with the epidermis averaging 0.03–0.04 mm in thickness. The total thickness of the facial subcutaneous tissue is 3–7 mm, including the superficial fat layer (approximately 1.5–3.5 mm thick), the SMAS (approximately 0.35–0.45 mm thick), the deep fat layer, and the deep fascia [76]. Therefore, the selection of appropriate photoelectric and ultrasonic devices and treatment parameters to target the changes in these tissue layers is crucial for achieving the goal of skin rejuvenation.

We summarize the mechanisms, levels, depths, and distribution patterns of thermal effects in skin remodeling using various energy devices (Table 1). Short or ultrashort pulse widths are typically employed for treating pigmented skin disorders, while long pulse widths (in the millisecond range) are used for vascular issues, and even longer pulse widths (millisecond to second range) are applied for tissue tightening and lifting. It is important to note that the treatment parameters of these energy devices—such as the power, frequency, pulse duration, and spot size—vary across studies. During treatment, physicians adjust these parameters based on their clinical experience and the skin's immediate response to achieve the most effective outcomes.

**TABLE 1:** Summary of thermal effects of energy device‐based skin resurfacing applications.

Device type	Energy modality	Wavelength/frequency	Target tissue	Mechanism	Thermal distribution	Clinical results
Light energy devices	CO_2_ laser	10 600 nm	Epidermis, middle dermis	Light energy converted to heat, water evaporation causes tissue ablation	Local evaporation and coagulation create micro‐damage zones. Ablation of approximately 100 μm of skin with incidental 50–300 μm of adjacent thermal damage.	Improved skin texture, reduced wrinkles, enhanced epidermal smoothness
	Er laser	2940 nm	Epidermis, superficial dermis	High water affinity, superficial tissue ablation	Superficial thermal damage, localized heat effect, ablates 4 μm per 1 J/mm^3^. Each pass causes tissue ablation of 20–40 μm and accompanying thermal damage of 5–30 μm	Reduction of fine lines, improved skin tightness
	Nd: YAG laser	1064 nm (long pulses; Q‐switched etc.) 1320 nm	Middle to deep dermis	Deep tissue heating, collagen remodeling, photomechanical effect	Deep thermal effect, induced collagen synthesis and tissue tightening, penetrates 1–2 mm, more than 4 mm	Reduced wrinkles, increased skin thickness
	Alexandrite laser	755 nm	Middle to deep dermis	Selective absorption by melanin and hemoglobin	Energy absorbed by chromophores generates thermal effect, penetrates up to 2–3 mm	Improved vascular lesions, pigmentation issues, and hair removal
	Pulsed dye laser	577/585/595 nm	Superficial dermis, blood vessels	Targets oxyhemoglobin, causes vascular coagulation	Selective thermal damage to superficial blood vessels, penetrates < 1.5 mm	Improved vascular lesions and pigmentation
	Nonablative fractional laser (NAFL)	1320–1927 nm	Deep dermis	Fractional heating creates microthermal zones (MTZs)	Thermal effect without epidermal damage, MTZs (< 100 μm) formation, penetrates into dermis (2–3 mm)	Reduced fine lines, improved scars, pigment reduction
	Intense pulsed light	500–1200 nm	Epidermis, superficial dermis	Selective photothermolysis of pigment and hemoglobin	Even thermal distribution, promotes superficial skin repair, penetrates 2 mm	Reduced pigmentation spots, improved redness, even skin tone
RF devices	Monopolar RF	3–40.5 MHz frequency, electrodes	Deep dermis, subcutaneous fat	Energy conducted through grounded electrode and skin electrode.	Deep and even thermal effect, penetrates 3–6 mm (half the size of the active electrode)	Skin tightening, wrinkle reduction, improved laxity
	Bipolar RF	3–40.5 MHz frequency, current between two electrodes	Epidermis, superficial dermis	Current between electrodes, with a fixed distance, while both electrodes are in contact to the skin.	More controlled and localized energy distribution pattern. Superficial thermal effect, penetrates 1–2 mm (half the distance between the electrodes)	Improved skin laxity and superficial wrinkles
Focused ultrasound devices	HIFU (high‐intensity focused ultrasound)	Frequency 2 MHz, energy 47–59 J/mm^2^	Sub‐dermis, SMAS layer	Mechanical vibration generates heat and cavitation effect	Strong heat focus, precisely distributed in tissue, penetrates 1.5–4.5 mm	Skin tightening, facial lifting, improved contour
	MFU (micro‐focused ultrasound)	Frequency 4–10 MHz, energy 0.4–1.2 J/mm^2^	Mid to deep dermis, SMAS layer	Mechanical energy creates thermal damage, stimulates dermis and SMAS	Micro‐damage to deep tissue promotes collagen production, depths of up to 5 mm	Significant lifting effect, wrinkle reduction, enhanced skin tightness
High‐intensity focused electromagnetic devices (HIFEM)	HIFES (high‐intensity facial electromagnetic stimulation)	< 10 kHz electromagnetic field	Muscle tissue, Subcutaneous fat layer	Electromagnetic energy stimulates muscle contraction, enhances muscle tone and firmness	Superficial and focused electromagnetic waves create muscle contractions, minimal heat penetration	Enhanced muscle tone, improved facial contour

### 3.1 Laser and Light Devices

**FIGURE 1:**
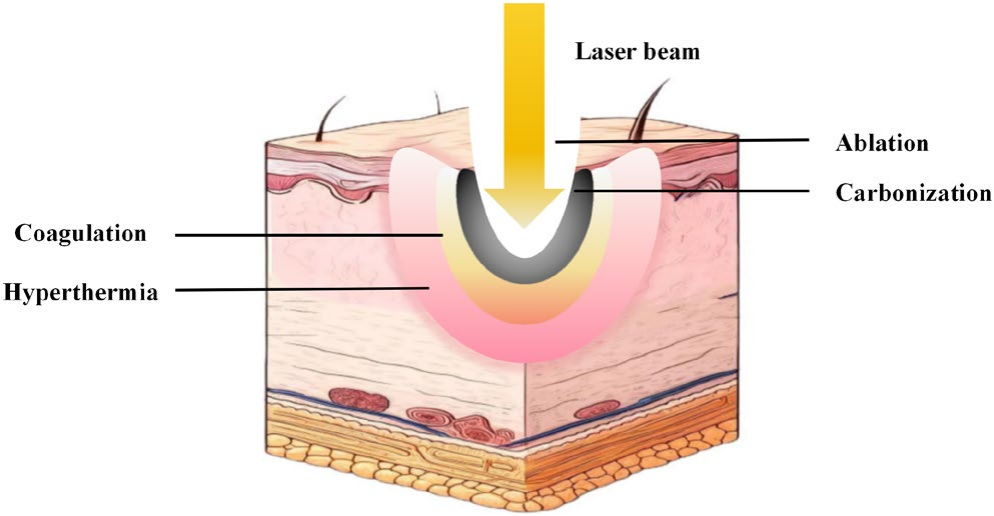
The laser produces a photothermal effect on the tissue, and different temperatures and their duration of action can cause different thermal effects on the tissue.

From the epidermis to the dermis, lasers can act at various wavelengths and strengths to cause a variety of biological effects at different levels (Figure 1). The earliest medical lasers were continuous‐wave lasers that removed skin from the entire operated area. However, their thermal dispersion could result in excessive damage to surrounding tissues due to the lack of precise control over the degree and extent of the thermal effect. Subsequently, quasi‐continuous wave lasers emerged, reducing the risk of thermal damage to some extent, but they remained nonspecific [77, 78, 79, 80]. Human tissues contain chromophores that can absorb visible or infrared light. Through selective absorption, lasers target specific tissue chromophores such as melanin, water, hemoglobin, and hydroxyapatite [81, 82, 83]. Choosing the appropriate wavelength enables effective targeting and determines the depth of light penetration. This maximizes the effect on the target tissue while minimizing the impact on surrounding structures [6, 28]. The theory of selective photothermolysis proposed by Anderson and Parrish (1983) [13] laid the foundation for a highly selective and safe laser.

The ablative laser can remove the epidermis, while the thermal effect can reach the middle part of the dermis, stimulating the remodeling of collagen. This process improves the skin texture and quality through the coagulative necrosis of epidermal cells and the tissue repair response in the dermis. Common types of ablative lasers include carbon dioxide (CO_2_) lasers and erbium‐yttrium‐aluminum‐garnet (Er:YAG) lasers. The 10 600 nm CO_2_ laser disrupts tissue by rapidly evaporating water, ablating approximately 100 μm of skin, along with adjacent thermal damage ranging from 50 to 300 μm. The Er:YAG laser has a high affinity for water, and the ablation depth of the short‐pulse Er:YAG laser is proportional to the total energy irradiated on the skin. For every 1 J/mm^3^, 4 μm of tissue can be ablated. Therefore, at general energy levels, only shallow thermal damage areas (5–20 μm) are left. Studies have shown that each pass causes tissue ablation of 20–40 μm and accompanying thermal damage of 5–30 μm [39, 84]. Nonablative lasers maintain the integrity of the epidermis, common ones such as the 585/595 nm pulsed dye laser (PDL), which targets oxygenated hemoglobin and acts mainly on the superficial layers of the skin; the 1320 nm neodymium‐doped yttrium aluminum garnet (Nd:YAG) laser penetrates well into the papillary dermis and the 1320‐nm neodymium‐doped yttrium aluminum garnet (Nd:YAG) laser penetrates well into the papillary dermis and the middle dermis, and is absorbed nonspecifically by the water in the dermis; the long‐pulsed 1064‐nm Nd:YAG laser's thermal effect area is 1–2 mm below the epidermis; the Q‐switched 1064‐nm Nd:YAG laser acts on the deeper dermis through an opto‐mechanical effect; the 755‐nm Alexandrite laser selectively affects the melanin and can penetrate up to 2 mm deep into the dermis; the thermal effect of the 1500–1600‐nm NAFL reaches the reticular layer of the dermis to a depth of up to 2–3 mm [11, 28, 85, 86, 87, 88, 89]. Laser technology has advanced significantly in the last decade, particularly with the introduction of the fractional laser treatment modality in 2004. This modality offers new treatment options for patients by inducing varying degrees of thermal effects on microdamage foci through either ablative or nonablative laser techniques. It enables the promotion of tissue remodeling within the microscopic treatment area [90, 91, 92, 93, 94, 95, 96].

Intense pulsed light (IPL) is a noncoherent light with broad wavelength coverage that passes through the epidermis while retaining its integrity [97]. It directly induces reversible thermal damage to the dermis and functions as a nonexfoliative light energy device. Despite not being a laser, the impact of this light source on tissue can be explained by the principle of selective photothermolysis. By selecting appropriate filters and adjusting the pulse width and the interval between pulses, a specific beam is directed to the target and targets specific chromophores [98, 99, 100, 101, 102, 103, 104, 105, 106].

As previously mentioned, the choice of wavelength primarily determines the depth of energy penetration. Furthermore, accurate management of the thermal effect necessitates a combination of appropriate pulse widths, energy fluxes, number of passes, handle movement rates, and spot sizes [18, 107, 108]. At a specific depth, the extent of temperature increase and thermal damage to the skin following laser treatment is directly linked to the laser dose, increasing with energy fluence and pulse width [18, 107, 108]. Second, in laser therapy, the thermal relaxation time is directly related to the target size. Spatial control of thermal damage, ranging from organ to organelle, can be achieved by using different exposure times, also called pulse widths. When the exposure time interval is less than the thermal relaxation time of the target chromophore, selective destruction of specific tissues can be achieved, reducing unnecessary thermal damage to surrounding tissues [13, 109, 110]. The pulse width also determines the mechanism of laser–tissue interaction. The long‐pulsed 1064 nm Nd:YAG laser primarily utilizes photothermal effects, while the Q‐switched 1064 nm laser primarily employs photomechanical effects [28]. In general, treatment devices can be selected based on the specific skin issues encountered. For instance, PDL systems offer varying wavelengths of laser bands (577, 585, and 595 nm) tailored to address vascular skin conditions and skin pigmentation. However, in practical application, the choice of wavelengths, pulse widths, and other parameters typically hinges on factors such as the nature of the skin problem, the patient's skin color, and the treatment area [111, 112, 113, 114]. The Fitzpatrick skin typing system is widely employed to classify skin into six grades (I–VI) based on its reaction to UV light, correlating with increasing melanin content. For example, types IV and VI have a higher melanin content and darker skin color. The epidermis has a wider absorption spectrum, absorbing more light energy and therefore producing relatively more heat. This has the potential to interfere with the depth of laser penetration and affect its efficacy. In practice, while higher energy levels may be indicated, doctors exercise caution to tailor laser parameters to avoid skin damage from excessive melanin absorption. This involves employing specific laser types, adjusting pulse widths, implementing enhanced cooling techniques, and employing other strategies to ensure the treatment's safety and efficacy [115, 116, 117, 118, 119, 120, 121, 122, 123, 124, 125]. For patients with darker skin tones, it is preferable to choose laser types that target water to avoid skin damage caused by excessive melanin absorption. In the case of hair removal on patients with fair skin and dark, dense hair, the long‐pulsed 755 nm laser that targets melanin is more suitable.

The addressing of various types of skin problems through light energy has become immensely popular, and light therapy based on these devices is increasingly prevalent in clinical practice. From continuous‐wave and quasi‐continuous‐wave lasers to pulsed and fractional lasers, all are progressing towards the concept of selective photothermolysis. This concept precisely controls the thermal effect on tissues, reducing excessive damage, shortening recovery time, and decreasing the incidence of associated complications. This advancement has improved the efficiency and safety of treatment while enhancing patient satisfaction.

### 3.2 Radiofrequency Energy Devices

**FIGURE 2:**
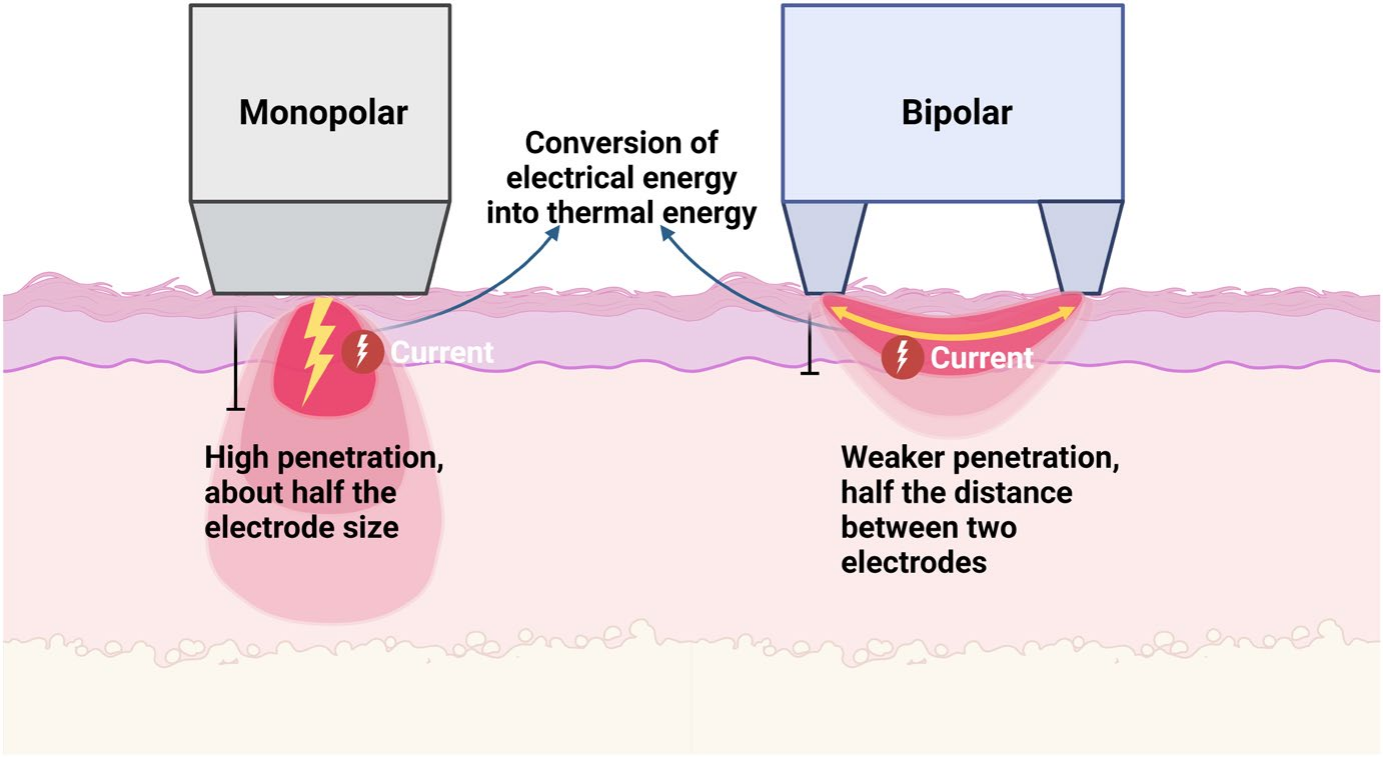
RF devices act on the tissues to convert electrical energy into thermal energy, inducing selective thermal damage to the skin. The main RF systems commonly used are monopolar and bipolar RF.

Radiofrequency (RF) refers to the frequency of oscillations per second of the electric and magnetic fields within the radio wave spectrum of the electromagnetic spectrum [126]. RF therapies use electrical waves in the frequency range of 3 MHz to 40.5 MHz. The intensity of RF penetration into the skin is inversely related to the frequency of the waves, which implies that the lower the frequency, the deeper the penetration of RF [35]. When RF energy is applied to the skin, it utilizes the electrical resistance within the skin layer or subcutaneous tissue to convert electrical energy into thermal energy. This process induces selective thermal skin damage, stimulating collagen neogenesis and rearrangement, resulting in tissue contraction and remodeling [127, 128, 129, 130, 131]. Unlike most lasers that target specific chromophores, RF relies on the electrical conductivity of the target tissue for selective electrothermolysis, eliminating the necessity for chromophores. RF energy primarily converts to heat through water within the tissue, rendering it unaffected by epidermal melanin and suitable for all skin types. The quantity of energy released as heat in the tissues is mostly determined by the RF generator's current intensity, tissue impedance, and duration of action. Water, nerves, muscle, collagen, other proteins, and fat are the order in which tissue resistance increases. Adipose tissue, for example, generates more heat due to its high impedance [33]. Monopolar and bipolar RF systems are the most commonly used in clinics. Monopolar RF concentrates electrical energy close to the electrode tip and penetrates to a depth that is about half the electrode's diameter. A deeper therapeutic depth (3–6 mm) is possible with monopolar devices due to their high current penetration, but at the expense of discomfort. Monopolar RF generates a diffuse current that operates on collagen in the dermis as well as a deeper network of fibrous compartments in the subcutaneous fat layer [132, 133]. In contrast, bipolar RF uses two electrodes that are placed over the treatment area and have current passing between them, which results in a depth of penetration approximately half the distance between them. While bipolar devices offer less penetration, they provide more controlled energy distribution and reduced discomfort (Figure 2). Additionally, bipolar RF devices are frequently combined with light‐based technology known as photodynamic synergy [126, 134, 135, 136, 137, 138, 139, 140, 141].

The desired depth of RF treatments varies according to specific treatment requirements. Optimal results hinge on selecting the appropriate treatment modality for different skin or tissue concerns. Typically, RF treatments excel at addressing skin laxity, fine lines, scar and acne. Conversely, deeper RF treatments target subcutaneous fat tissues, aiming for localized fat reduction and body contouring through the heating of fat cells and stimulation of collagen regeneration [137, 142, 143, 144]. The latest RF devices use fractional technology to deliver RF energy through an array of electrodes, or microneedles. This selectively heats the contact area while keeping the surrounding area intact [145, 146, 147, 148, 149, 150]. Additionally, Wilczyński et al. [35] utilized dynamic thermography to investigate skin temperature variations in different anatomical locations subjected to RF action. They showed that applying the same treatment parameters to different anatomical locations may result in distinct thermal responses. Specifically, after RF treatment, the peak skin temperature is higher in areas with a thicker adipose tissue layer. Conversely, in regions with less adipose tissue, patients have a lower tolerance to RF treatment at the same parameters. Therefore, in order to achieve safe and effective RF treatment, we need to consider the type of RF equipment, frequency, energy, duration of action, and individual patient variability.

### 3.3 Focused Ultrasound Energy Devices

**FIGURE 3:**
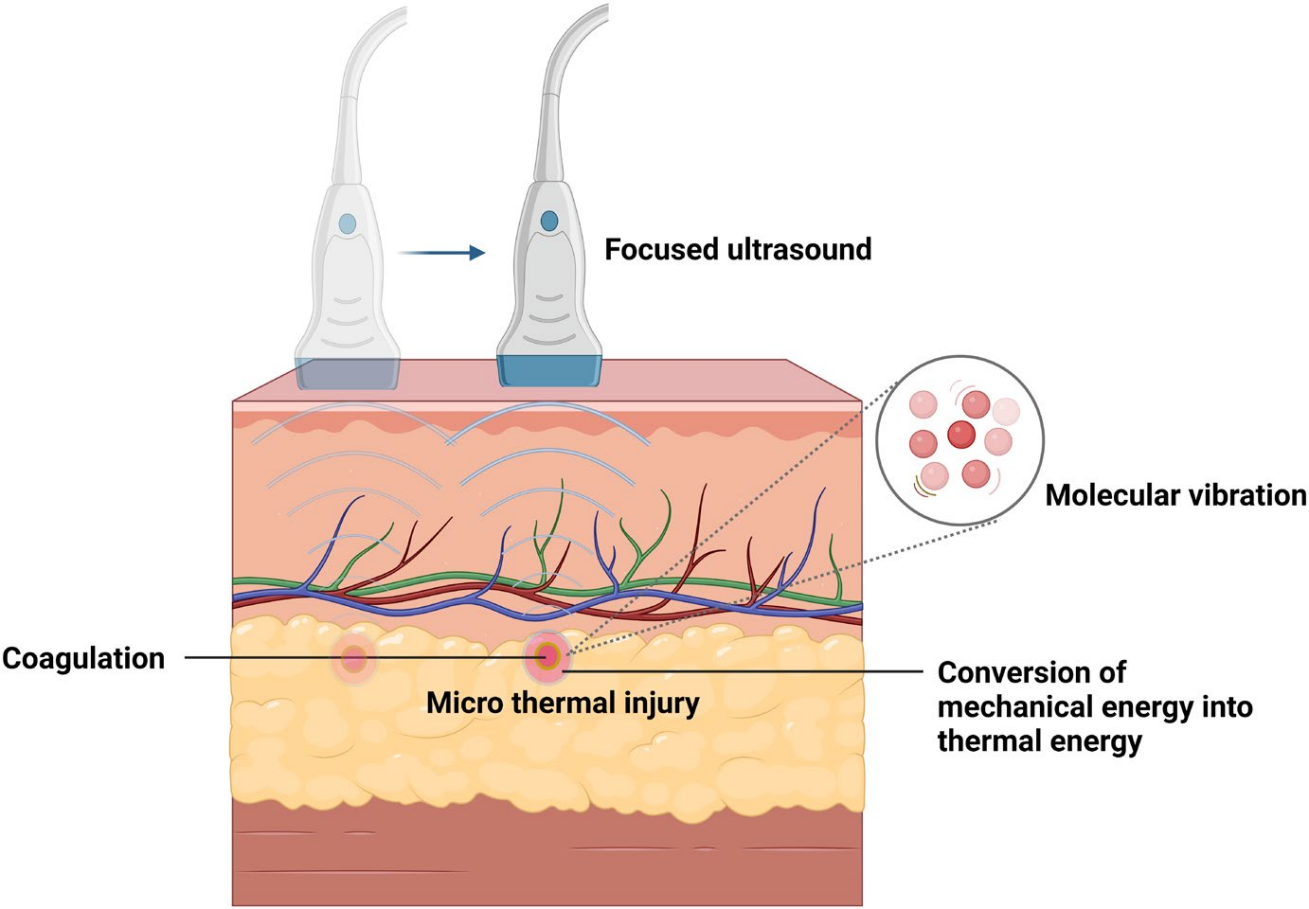
Focused ultrasound energy beams act precisely on the tissue at the level of the focal point. The tissue absorbs the ultrasound energy causing molecular vibrations, converting mechanical energy into thermal energy and producing immediate micro‐thermal damage.

Focused ultrasonic energy equipment uses high‐frequency sound waves above the hearing threshold to cause molecular vibration, which can induce heat and cavitation effects, resulting in cell damage and death. Focused ultrasound allows precise targeting of tissue, producing a small, well‐defined, controlled zone of thermal damage with minimal damage to surrounding structures [41, 151]. Different frequencies, depths of focus, and energy levels can be used to create highly selective action areas of depth and size [152]. The two main types of focused ultrasound used in medicine are microfocused ultrasound and high‐intensity focused ultrasound. High‐intensity focused ultrasound (HIFU) employs high‐frequency sound waves, primarily applied in medical aesthetics for ablating fatty tissues and noninvasively contouring the body. Conversely, microfocused ultrasound (MFU) utilizes lower levels of ultrasound energy to target the superficial layers of the skin, effectively achieving skin tightening [153, 154, 155]. HIFU energy beams, characterized by their high frequency, can be precisely directed to pass harmlessly through the skin and act on targeted tissues such as fat tissue and the superficial musculoskeletal nervous system [156, 157]. The tissue absorbs the ultrasound energy, causing molecular vibration, converting mechanical energy into thermal energy, and producing immediate micro‐thermal damage. This triggers thermal coagulation of the tissue in a specific area [34, 158]. Simultaneously, the propagation of ultrasonic waves through tissues involves repetitive compression and rarefaction, which results in strong shear forces. This shearing motion generates frictional heat while simultaneously causing small bubbles in biological tissue fluids to swell and oscillate until they collapse. The collapse of these bubbles produces high temperatures within, which leads to cell death via mechanical processes caused by the forces produced during collapse [41] (Figure 3). MFU is mostly utilized for skin tightening and wrinkle reduction. The most generally utilized treatment parameters are 0.4–1.2 J/mm^2^ energy, 4–10 MHz frequency, and 1.5–4.5 mm depth of focus, which can be focused on and in the deep reticular layer of the dermis and subcutaneous tissues while leaving the papillary and epidermal layers undisturbed. The papillary dermis and epidermis remain unaffected. Current transducers emit at 10.0, 7.0, and 4.0 MHz, with focal depths of 1.5 mm (dermis), 3.0 mm (deep dermis), and 4.5 mm (subcutaneous tissues, including the SMAS layer). Clinically, HIFU uses 47–59 J/cm^2^ of energy, a frequency of approximately 2 MHz, and a depth of focus of 1.1–1.8 cm to ablate subcutaneous fat [34, 41, 76, 122].

### 3.4 High‐Intensity Focused Electromagnetic Devices

High‐intensity focused electromagnetic technology (HIFEM) uses low‐frequency electromagnetic fields of < 10 kHz to activate neurons in skeletal muscles, resulting in intense involuntary muscular contractions [159, 160]. This stimulation causes increased metabolism, raised extracellular fatty acid levels, adipocyte malfunction and apoptosis due to endoplasmic reticulum stress, and new muscle fiber formation by muscle satellite cells, all of which result in myofibrillar hyperplasia and fat loss [157, 161, 162, 163, 164]. The combination of HIFEM and RF has been found to be safe and effective in fat loss and muscle strengthening, reducing treatment time and enhancing outcomes [165, 166]. The recently created high‐intensity facial electromagnetic stimulation (HIFES) system, which is synchronized with RF technology, was designed exclusively to combat facial aging [167].

HIFES uses electromagnetic pulses to activate small superficial facial muscles, such as the frontalis and zygomaticus, restoring muscle tone, increasing muscle volume, tightening fascia, and remodeling the skin for a volumizing effect. It can also reduce dynamic wrinkles caused by excessive expression muscle movement by strengthening antagonist muscles or weakening dynamic muscle fibers [168, 169]. Kinney et al. [167] used RF in combination with HIFES to treat the porcine frontalis muscle, and the follow‐up results revealed a significant increase in facial muscle area, density, and mass, as well as histologic evidence of progressive muscle tissue replacement of adipose and connective tissue. Through simultaneous RF and HIFES technology, the tissue is heated within the effective temperature range to promote collagen regeneration and electrical stimulation of specific facial muscles, resulting in noninvasive face lifting, wrinkle reduction, facial contour reshaping, and skin texture improvement [170, 171].

## 4 Disscussion

Energy‐based devices convert thermal energy and transfer it to the target area using various forms of energy like light, focused ultrasound, radiofrequency, etc. This induces a thermal effect on the tissues, treating and repairing the skin for regeneration and remodeling. Utilizing photoelectric and ultrasonic technology for skin rejuvenation involves a rational application of tissue fibrosis. Fibrosis is the process involving collagen synthesis, deposition, and extracellular matrix reconstruction in damaged tissues during tissue repair or pathology. It is a crucial biological process that significantly contributes to enhancing the elasticity, density, and texture of the skin. It serves as the foundation for skin tissue repair and regeneration, yet it is a double‐edged sword.

The primary objective of using energy devices in skin rejuvenation is to achieve a controlled and beneficial thermal effect within the dermis and subcutaneous fascia layers while minimizing damage to the epidermis. It is important to consider the threshold of irreversible thermal damage that affects different components of the skin tissue. Within a certain range, a higher intensity of thermal stimulation results in a more favorable thermal effect. Reversible thermal damage and, to a lesser extent or at shallower depths in the skin, irreversible thermal damage can increase the metabolic activity of cells in the treated area or in adjacent tissues. This stimulation promotes the synthesis and deposition of collagen during the repair process, filling in damaged tissues and improving the strength and stability of the skin. However, if irreversible thermal injury is too severe, it can lead to challenging tissue damage, apoptosis (programmed cell death), and excessive necrosis. The subsequent excessive fibrosis can negatively affect the tissues, causing tightness, loss of natural softness and elasticity, and potentially resulting in irregular texture and scar formation on the skin surface. It is important to carefully manage the interval between treatments to allow the skin to recover and generate new collagen after each session. The optimal treatment interval should be determined based on individual skin response, treatment goals, and the specific treatment area to avoid excessive fibrosis.

## 5 Conclusion

The thermal effect is critical when using photoelectric and ultrasonic technologies in skin remodeling. Thermal damage and rejuvenating benefits can be assessed by examining tissue changes at various temperatures. Analyzing molecular markers is critical for tracking injuries and avoiding serious harm. Understanding device processes, treatment settings, and patient variances is essential for controlling thermal effects. Optimizing these characteristics results in more effective outcomes while lowering hazards. Continuous monitoring and research will help us better manage heat damage in skin treatments.

## Author Contributions

Ximeng Jia collected the data and wrote the paper. Yongqiang Feng developed the idea and assisted the first author to revise and polish the paper. All authors contributed to the article and approved the submitted version.

## Ethics Statement

All procedures performed in studies involving human participants were in accordance with the institutional and/or national research committee's ethical standards, as well as the 1964 Helsinki Declaration and its subsequent amendments or comparable ethical standards.

## Conflicts of Interest

The authors declare no conflicts of interest.

## Data Availability

Data sharing not applicable to this article as no datasets were generated or analysed during the current study.

## References

[1] S. Nassar , M. Assem , D. Mohamed , and G. Hassan , “The Efficacy of Radiofrequency, Intense Pulsed Light and Carboxytherapy in Facial Rejuvenation,” Journal of Cosmetic and Laser Therapy 22, no. 6–8 (2020): 256–264. 33840336 10.1080/14764172.2021.1880598

[2] M. el‐Domyati , T. el‐Ammawi , W. Medhat , et al., “Radiofrequency Facial Rejuvenation: Evidence‐Based Effect,” Journal of the American Academy of Dermatology 64, no. 3 (2011): 524–535. 21315951 10.1016/j.jaad.2010.06.045 PMC6541915

[3] G. Magni , L. Pieri , I. Fusco , F. Madeddu , T. Zingoni , and F. Rossi , “Laser Emission at 675 nm: In Vitro Study Evidence of a Promising Role in Skin Rejuvenation,” Regenerative Therapy 22 (2023): 176–180. 36819611 10.1016/j.reth.2023.01.007 PMC9930151

[4] M. Alam , T. S. Hsu , J. S. Dover , D. A. Wrone , and K. A. Arndt , “Nonablative Laser and Light Treatments: Histology and Tissue Effects—A Review,” Lasers in Surgery and Medicine 33, no. 1 (2003): 30–39. 12866119 10.1002/lsm.10195

[5] M. El‐Domyati , T. Abd‐El‐Raheem , W. Medhat , H. Abdel‐Wahab , and A. M. Al , “Multiple Fractional Erbium: Yttrium‐Aluminum‐Garnet Laser Sessions for Upper Facial Rejuvenation: Clinical and Histological Implications and Expectations,” Journal of Cosmetic Dermatology 13, no. 1 (2014): 30–37. 24641603 10.1111/jocd.12079

[6] A. J. González‐Rodríguez and R. Lorente‐Gual , “Current Indications and New Applications of Intense Pulsed Light,” Actas Dermo‐Sifiliográficas 106, no. 5 (2015): 350–364. 25638325 10.1016/j.ad.2014.10.004

[7] D. Li , H. Zhang , B. Chen , et al., “Experimental Investigations on Thermal Effects of a Long‐Pulse Alexandrite Laser on Blood Vessels and Its Comparison With Pulsed Dye and Nd:YAG Lasers,” Lasers in Medical Science 35, no. 7 (2020): 1555–1566. 32060655 10.1007/s10103-020-02981-9

[8] Y. Shi , W. Jiang , W. Li , W. Zhang , and Y. Zou , “Comparison of Fractionated Frequency‐Doubled 1,064/532 nm Picosecond Nd:YAG Lasers and Non‐Ablative Fractional 1,540 nm Er: Glass in the Treatment of Facial Atrophic Scars: A Randomized, Split‐Face, Double‐Blind Trial,” Annals of Translational Medicine 9, no. 10 (2021): 862. 34164496 10.21037/atm-21-1715 PMC8184496

[9] Y. Rhee Do , H. I. Cho , G. H. Park , et al., “Histological and Molecular Analysis of the Long‐Pulse 1,064‐nm Nd:YAG Laser Irradiation on the Ultraviolet‐Damaged Skin of Hairless Mice: In Association With Pulse Duration Change,” Journal of Cosmetic and Laser Therapy 18, no. 1 (2016): 16–21. 26052812 10.3109/14764172.2015.1052509

[10] P. G. Ruff, 4th , “Thermal Effects of Percutaneous Application of Plasma/Radiofrequency Energy on Porcine Dermis and Fibroseptal Network,” Journal of Cosmetic Dermatology 20, no. 7 (2021): 2125–2131. 33197275 10.1111/jocd.13845 PMC8359425

[11] S. P. Nisticò , L. Bennardo , T. Zingoni , et al., “Synergistic Sequential Emission of Fractional 10.600 and 1540 nm Lasers for Skin Resurfacing: An Ex Vivo Histological Evaluation,” Medicina (Kaunas, Lithuania) 58, no. 9 (2022): 1308. doi:10.3390/medicina58091308. 36143985 PMC9502429

[12] J. W. Byun , Y. R. Kang , S. Park , and W. Hong , “Efficacy of Radiofrequency Combined With Single‐Dot Ultrasound Efficacy for Skin Rejuvenation: A Non‐Randomized Split‐Face Trial With Blinded Response Evaluation,” Skin Research and Technology 29, no. 9 (2023): e13452. 37753689 10.1111/srt.13452 PMC10496459

[13] R. R. Anderson and J. A. Parrish , “Selective Photothermolysis: Precise Microsurgery by Selective Absorption of Pulsed Radiation,” Science 220, no. 4596 (1983): 524–527. 6836297 10.1126/science.6836297

[14] A. Capon and S. Mordon , “Can Thermal Lasers Promote Skin Wound Healing,” American Journal of Clinical Dermatology 4, no. 1 (2003): 1–12. 12477368 10.2165/00128071-200304010-00001

[15] C. J. Chang , D. Y. Yu , Y. C. Hsiao , and K. H. Ho , “Noninvasive Imaging Analysis of Biological Tissue Associated With Laser Thermal Injury,” Biomedical Journal 40, no. 2 (2017): 106–112. 28521901 10.1016/j.bj.2016.10.004 PMC6138599

[16] M. Wang‐Evers , A. J. Blazon‐Brown , L. Ha‐Wissel , et al., “Assessment of a 3050/3200 nm Fiber Laser System for Ablative Fractional Laser Treatments in Dermatology,” Lasers in Surgery and Medicine 54, no. 6 (2022): 851–860. 35395696 10.1002/lsm.23550 PMC9541207

[17] J. K. Duplechain , “Ablative Laser Therapy of Skin,” Facial Plastic Surgery Clinics of North America 31, no. 4 (2023): 463–473. 37806680 10.1016/j.fsc.2023.05.002

[18] J. C. Shen , Y. Y. Liang , and W. Li , “Quantitative Simulation of Photothermal Effect in Laser Therapy of Hypertrophic Scar,” Skin Research and Technology 29, no. 3 (2023): e13305. 36973985 10.1111/srt.13305 PMC10155850

[19] J. T. Beckham , M. A. Mackanos , C. Crooke , et al., “Assessment of Cellular Response to Thermal Laser Injury Through Bioluminescence Imaging of Heat Shock Protein 70,” Photochemistry and Photobiology 79, no. 1 (2004): 76–85. 14974719

[20] M. S. Alayat , A. M. Elsodany , A. F. Miyajan , A. A. Alzhrani , H. Alzhrani , and A. M. Maqliyah , “Changes in Local Skin Temperature After the Application of a Pulsed Nd:YAG Laser to Healthy Subjects: A Prospective Crossover Controlled Trial,” Lasers in Medical Science 34, no. 8 (2019): 1681–1688. 30903525 10.1007/s10103-019-02769-6

[21] B. Winship , D. Wollin , E. Carlos , et al., “The Rise and Fall of High Temperatures During Ureteroscopic Holmium Laser Lithotripsy,” Journal of Endourology 33, no. 10 (2019): 794–799. 31016991 10.1089/end.2019.0084

[22] S. A. Brown , J. P. Farkas , C. Arnold , et al., “Heat Shock Proteins 47 and 70 Expression in Rodent Skin Model as a Function of Contact Cooling Temperature: Are We Overcooling Our Target,” Lasers in Surgery and Medicine 39, no. 6 (2007): 504–512. 17659589 10.1002/lsm.20517

[23] K. Y. Kung , S. Y. Shek , C. K. Yeung , and H. H. Chan , “Evaluation of the Safety and Efficacy of the Dual Wavelength Picosecond Laser for the Treatment of Benign Pigmented Lesions in Asians,” Lasers in Surgery and Medicine 51, no. 1 (2019): 14–22. 30357871 10.1002/lsm.23028

[24] M. Al‐Muriesh , C. Z. Huang , Z. Ye , and J. Yang , “Dermoscopy and VISIA Imager Evaluations of Non‐Insulated Microneedle Radiofrequency Versus Fractional CO(2) Laser Treatments of Striae Distensae,” Journal of the European Academy of Dermatology and Venereology 34, no. 8 (2020): 1859–1866. 32030833 10.1111/jdv.16266

[25] S. Hu , C. S. Yang , S. L. Chang , Y. L. Huang , Y. F. Lin , and M. C. Lee , “Efficacy and Safety of the Picosecond 755‐nm Alexandrite Laser for Treatment of Dermal Pigmentation in Asians‐a Retrospective Study,” Lasers in Medical Science 35, no. 6 (2020): 1377–1383. 31965352 10.1007/s10103-020-02959-7

[26] N. M. Williams , P. Gurnani , J. Long , et al., “Comparing the Efficacy and Safety of Q‐Switched and Picosecond Lasers in the Treatment of Nevus of Ota: A Systematic Review and Meta‐Analysis,” Lasers in Medical Science 36, no. 4 (2021): 723–733. 32839837 10.1007/s10103-020-03125-9

[27] N. Dorgham , B. Witkoff , E. Weiss , and B. Glick , “Severe Persistent Urticaria Following Laser Hair Reduction,” Journal of Cosmetic Dermatology 21, no. 12 (2022): 6698–6701. 36106510 10.1111/jocd.15390

[28] H. Liu , Y. Dang , Z. Wang , X. Chai , and Q. Ren , “Laser Induced Collagen Remodeling: A Comparative Study In Vivo on Mouse Model,” Lasers in Surgery and Medicine 40, no. 1 (2008): 13–19. 18220261 10.1002/lsm.20587

[29] F. Hiragami , H. Motoda , T. Takezawa , et al., “Heat Shock‐Induced Three‐Dimensional‐Like Proliferation of Normal Human Fibroblasts Mediated by Pressed Silk,” International Journal of Molecular Sciences 10, no. 11 (2009): 4963–4976. 20087471 10.3390/ijms10114963 PMC2808017

[30] S. D. Dams , B. M. de Liefde‐van , A. M. Nuijs , C. W. Oomens , and F. P. Baaijens , “Pulsed Heat Shocks Enhance Procollagen Type I and Procollagen Type III Expression in Human Dermal Fibroblasts,” Skin Research and Technology 16, no. 3 (2010): 354–364. 20637005 10.1111/j.1600-0846.2010.00441.x

[31] S. D. Dams , B. M. de Liefde‐van , A. M. Nuijs , C. W. Oomens , and F. P. Baaijens , “Heat Shocks Enhance Procollagen Type I and III Expression in Fibroblasts in Ex Vivo Human Skin,” Skin Research and Technology 17, no. 2 (2011): 167–180. 21251083 10.1111/j.1600-0846.2010.00473.x

[32] H. Grema , B. Greve , and C. Raulin , “Facial Rhytides—Subsurfacing or Resurfacing? A Review,” Lasers in Surgery and Medicine 32, no. 5 (2003): 405–412. 12766965 10.1002/lsm.10172

[33] R. D. Gentile , B. M. Kinney , and N. S. Sadick , “Radiofrequency Technology in Face and Neck Rejuvenation,” Facial Plastic Surgery Clinics of North America 26, no. 2 (2018): 123–134. 29636146 10.1016/j.fsc.2017.12.003

[34] M. Juhász , D. Korta , and N. A. Mesinkovska , “A Review of the Use of Ultrasound for Skin Tightening, Body Contouring, and Cellulite Reduction in Dermatology,” Dermatologic Surgery 44, no. 7 (2018): 949–963. 29846343 10.1097/DSS.0000000000001551

[35] S. Wilczyński , A. Stolecka‐Warzecha , A. Deda , et al., “In Vivo Dynamic Thermal Imaging of Skin Radiofrequency Treatment,” Journal of Cosmetic Dermatology 18, no. 5 (2019): 1307–1316. 30225860 10.1111/jocd.12775

[36] R. Saluja and R. D. Gentile , “Picosecond Laser: Tattoos and Skin Rejuvenation,” Facial Plastic Surgery Clinics of North America 28, no. 1 (2020): 87–100. 31779945 10.1016/j.fsc.2019.09.008

[37] S. J. de Filippi , T. H. Osaki , M. H. Osaki , R. B. de Souza , and N. Allemann , “‘Split‐Face’ Evaluation of Collagen Changes Induced by Periorbital Fractional CO_2_ Laser Resurfacing,” Aesthetic Surgery Journal 42, no. 3 (2022): 239–248. 34618888 10.1093/asj/sjab357

[38] R. E. Fitzpatrick , E. F. Rostan , and N. Marchell , “Collagen Tightening Induced by Carbon Dioxide Laser Versus Erbium: YAG Laser,” Lasers in Surgery and Medicine 27, no. 5 (2000): 395–403. 11126433 10.1002/1096-9101(2000)27:5<395::AID-LSM1000>3.0.CO;2-4

[39] D. J. Goldberg , “Lasers for Facial Rejuvenation,” American Journal of Clinical Dermatology 4, no. 4 (2003): 225–234. 12680801 10.2165/00128071-200304040-00002

[40] M. Majidian , H. Kolli , and R. L. Moy , “Management of Skin Thinning and Aging: Review of Therapies for Neocollagenesis; Hormones and Energy Devices,” International Journal of Dermatology 60, no. 12 (2021): 1481–1487. 33739464 10.1111/ijd.15541

[41] S. G. Fabi , “Noninvasive Skin Tightening: Focus on New Ultrasound Techniques,” Clinical, Cosmetic and Investigational Dermatology 8 (2015): 47–52. 25709486 10.2147/CCID.S69118 PMC4327394

[42] T. J. Lee , D. Kim , T. Kim , C. J. Pak , H. P. Suh , and J. P. Hong , “Rejuvenation of Photoaged Aged Mouse Skin Using High‐Intensity Focused Ultrasound,” Journal of Plastic, Reconstructive & Aesthetic Surgery 75, no. 10 (2022): 3859–3868. 10.1016/j.bjps.2022.06.073 36041975

[43] M. Muddassir , G. Limbert , and D. Navarro‐Alarcon , “Development of a Numerical Multi‐Layer Model of Skin Subjected to Pulsed Laser Irradiation to Optimise Thermal Stimulation in Photorejuvenation Procedure,” Computer Methods and Programs in Biomedicine 216 (2022): 106653. 35144148 10.1016/j.cmpb.2022.106653

[44] E. Cuerda‐Galindo , G. Díaz‐Gil , M. A. Palomar‐Gallego , and R. Linares‐GarcíaValdecasas , “Intense Pulsed Light Induces Synthesis of Dermal Extracellular Proteins In Vitro,” Lasers in Medical Science 30, no. 7 (2015): 1931–1939. 26188855 10.1007/s10103-015-1787-5

[45] W. Sun , C. Zhang , J. Zhao , J. Wu , and L. Xiang , “Comparison of Moderate and High Energy of a Nano‐Fractional Radiofrequency Treatment on a Photoaging Hairless Mice Model,” Dermatologic Surgery 44, no. 4 (2018): 569–575. 29053534 10.1097/DSS.0000000000001362

[46] R. I. Morimoto , “Cells in Stress: Transcriptional Activation of Heat Shock Genes,” Science 259 (1993): 1409–1410, 10.1126/science.8451637. 8451637

[47] A. K. Verrico and J. V. Moore , “Expression of the Collagen‐Related Heat Shock Protein HSP47 in Fibroblasts Treated With Hyperthermia or Photodynamic Therapy,” British Journal of Cancer 76 (1997): 719–724, 10.1038/bjc.1997.452. 9310236 PMC2228048

[48] B. M. Hantash , V. P. Bedi , B. Kapadia , et al., “In Vivo Histological Evaluation of a Novel Ablative Fractional Resurfacing Device,” Lasers in Surgery and Medicine 39, no. 2 (2007): 96–107. 17311274 10.1002/lsm.20468

[49] M. Dehbi , E. Baturcam , A. Eldali , et al., “Hsp‐72, a Candidate Prognostic Indicator of Heatstroke,” Cell Stress & Chaperones 15 (2010): 593–603, 10.1007/s12192-010-0172-3. 20174993 PMC3006628

[50] A. Y. Sajjadi , K. Mitra , and M. Grace , “Expression of Heat Shock Proteins 70 and 47 in Tissues Following Short‐Pulse Laser Irradiation: Assessment of Thermal Damage and Healing,” Medical Engineering & Physics 35 (2013): 1406–1414, 10.1016/j.medengphy.2013.03.011. 23587755

[51] S. Tang , R. Buriro , Z. Liu , et al., “Localization and Expression of Hsp27 and αB‐Crystallin in Rat Primary Myocardial Cells During Heat Stress In Vitro,” PLoS One 8 (2013): e69066, 10.1371/journal.pone.0069066. 23894407 PMC3716771

[52] K. E. Karmisholt , E. Wenande , D. Thaysen‐Petersen , P. A. Philipsen , U. Paasch , and M. Haedersdal , “Early Intervention With Non‐Ablative Fractional Laser to Improve Cutaneous Scarring—A Randomized Controlled Trial on the Impact of Intervention Time and Fluence Levels,” Lasers in Surgery and Medicine 50 (2018): 28–36, 10.1002/lsm.22707. 28815643

[53] L. Calapre , E. S. Gray , and M. Ziman , “Heat Stress: A Risk Factor for Skin Carcinogenesis,” Cancer Letters 337 (2013): 35–40, 10.1016/j.canlet.2013.05.039. 23748013

[54] R. E. Barrow and M. R. Dasu , “Oxidative and Heat Stress Gene Changes in Hypertrophic Scar Fibroblasts Stimulated With Interleukin‐1Beta,” Journal of Surgical Research 126 (2005): 59–65, 10.1016/j.jss.2005.01.011. 15916976

[55] S. A. Abdelnour , M. E. Abd El‐Hack , A. F. Khafaga , M. Arif , A. E. Taha , and A. E. Noreldin , “Stress Biomarkers and Proteomics Alteration to Thermal Stress in Ruminants: A Review,” Journal of Thermal Biology 79 (2019): 120–134, 10.1016/j.jtherbio.2018.12.013. 30612672

[56] W. Huang , W. Xie , J. Gong , et al., “Heat Stress Induces RIP1/RIP3‐Dependent Necroptosis Through the MAPK, NF‐κB, and c‐Jun Signaling Pathways in Pulmonary Vascular Endothelial Cells,” Biochemical and Biophysical Research Communications 528 (2020): 206–212, 10.1016/j.bbrc.2020.04.150. 32471717

[57] S. Chen , Y. Yong , and X. Ju , “Effect of Heat Stress on Growth and Production Performance of Livestock and Poultry: Mechanism to Prevention,” Journal of Thermal Biology 99 (2021): 103019, 10.1016/j.jtherbio.2021.103019. 34420644

[58] S. Banerjee Mustafi , P. K. Chakraborty , R. S. Dey , and S. Raha , “Heat Stress Upregulates Chaperone Heat Shock Protein 70 and Antioxidant Manganese Superoxide Dismutase Through Reactive Oxygen Species (ROS), p38MAPK, and Akt,” Cell Stress & Chaperones 14 (2009): 579–589, 10.1007/s12192-009-0109-x. 19291423 PMC2866949

[59] A. Mahanty , S. Mohanty , and B. P. Mohanty , “Dietary Supplementation of Curcumin Augments Heat Stress Tolerance Through Upregulation of Nrf‐2‐Mediated Antioxidative Enzymes and Hsps in Puntius Sophore,” Fish Physiology and Biochemistry 43 (2017): 1131–1141, 10.1007/s10695-017-0358-z. 28315162

[60] K. Srikanth , A. Kwon , E. Lee , and H. Chung , “Characterization of Genes and Pathways That Respond to Heat Stress in Holstein Calves Through Transcriptome Analysis,” Cell Stress & Chaperones 22 (2017): 29–42, 10.1007/s12192-016-0739-8. 27848120 PMC5225057

[61] C. Wang , Y. L. Zhou , Q. H. Zhu , et al., “Effects of Heat Stress on the Liver of the Chinese Giant Salamander Andrias Davidianus: Histopathological Changes and Expression Characterization of Nrf2‐Mediated Antioxidant Pathway Genes,” Journal of Thermal Biology 76 (2018): 115–125, 10.1016/j.jtherbio.2018.07.016. 30143286

[62] C. He , J. Sun , D. Yang , et al., “Nrf2 Activation Mediates the Protection of Mouse Sertoli Cells Damage Under Acute Heat Stress Conditions,” Theriogenology 177 (2022): 183–194, 10.1016/j.theriogenology.2021.10.009. 34715543

[63] S. E. Tran , A. Meinander , T. H. Holmström , et al., “Heat Stress Downregulates FLIP and Sensitizes Cells to Fas Receptor‐Mediated Apoptosis,” Cell Death and Differentiation 10 (2003): 1137–1147, 10.1038/sj.cdd.4401278. 14502237

[64] S. Chinnathambi , A. Tomanek‐Chalkley , and J. R. Bickenbach , “HSP70 and EndoG Modulate Cell Death by Heat in Human Skin Keratinocytes In Vitro,” Cells, Tissues, Organs 187 (2008): 131–140, 10.1159/000109941. 17938562

[65] C. Zhang , J. Dai , Y. Fan , X. He , and R. Wei , “MicroRNA‐16 Inhibits Cell Proliferation and Migration by Targeting Heat Shock Protein 70 in Heat‐Denatured Dermal Fibroblasts,” Korean Journal of Internal Medicine 34 (2019): 634–642, 10.3904/kjim.2016.315. 29294597 PMC6506753

[66] X. H. Zhang , J. X. Wu , J. Z. Sha , B. Yang , J. R. Sun , and E. D. Bao , “Heat Shock Protein 90 Relieves Heat Stress Damage of Myocardial Cells by Regulating Akt and PKM2 Signaling In Vivo,” International Journal of Molecular Medicine 45 (2020): 1888–1908, 10.3892/ijmm.2020.4560. 32236591 PMC7169958

[67] H. O. Rennekampff and Z. Alharbi , “Burn Injury: Mechanisms of Keratinocyte Cell Death,” Medical Sciences (Basel, Switzerland) 9 (2021): 51, 10.3390/medsci9030051. 34287312 PMC8293431

[68] S. Zhu , Z. Yang , L. Kong , L. Kong , and Y. Zhang , “Arbutin Inhibited Heat Stress‐Induced Apoptosis and Promoted Proliferation and Migration of Heat‐Injured Dermal Fibroblasts and Keratinocytes by Activating PI3K/AKT Signaling Pathway,” Evidence‐Based Complementary and Alternative Medicine 2022 (2022): 8798861, 10.1155/2022/8798861. 36159569 PMC9499752

[69] J. Lu , H. Li , D. Yu , P. Zhao , and Y. Liu , “Heat Stress Inhibits the Proliferation and Differentiation of Myoblasts and Is Associated With Damage to Mitochondria,” Frontiers in Cell and Development Biology 11 (2023): 1171506, 10.3389/fcell.2023.1171506. PMC10126414 37113771

[70] B. Zhang , M. Wang , A. Gong , et al., “HucMSC‐Exosome Mediated‐Wnt4 Signaling Is Required for Cutaneous Wound Healing,” Stem Cells 33 (2015): 2158–2168, 10.1002/stem.1771. 24964196

[71] T. Zhang , X. F. Wang , Z. C. Wang , et al., “Current Potential Therapeutic Strategies Targeting the TGF‐β/Smad Signaling Pathway to Attenuate Keloid and Hypertrophic Scar Formation,” Biomedicine & Pharmacotherapy 129 (2020): 110287, 10.1016/j.biopha.2020.110287. 32540643

[72] J. Y. Zhou , D. G. Huang , M. Zhu , et al., “Wnt/β‐Catenin‐Mediated Heat Exposure Inhibits Intestinal Epithelial Cell Proliferation and Stem Cell Expansion Through Endoplasmic Reticulum Stress,” Journal of Cellular Physiology 235 (2020): 5613–5627, 10.1002/jcp.29492. 31960439

[73] K. J. Ahn and J. S. Kim , “TGF‐β1 Upregulates Sar1a Expression and Induces Procollagen‐I Secretion in Hypertrophic Scarring Fibroblasts,” Open Medicine (Warsaw, Poland) 17 (2022): 1473–1482, 10.1515/med-2022-0543. 36188194 PMC9483117

[74] G. Ge , Y. Wang , Y. Xu , et al., “Induced Skin Aging by Blue‐Light Irradiation in Human Skin Fibroblasts via TGF‐β, JNK and EGFR Pathways,” Journal of Dermatological Science 111 (2023): 52–59, 10.1016/j.jdermsci.2023.06.007. 37438186

[75] X. Li , R. Xie , Y. Luo , et al., “Cooperation of TGF‐β and FGF Signalling Pathways in Skin Development,” Cell Proliferation 56 (2023): e13489, 10.1111/cpr.13489. 37150846 PMC10623945

[76] H. S. Lee , W. S. Jang , Y. J. Cha , et al., “Multiple Pass Ultrasound Tightening of Skin Laxity of the Lower Face and Neck,” Dermatologic Surgery 38 (2012): 20–27, 10.1111/j.1524-4725.2011.02158.x. 22092848

[77] C. A. Nanni and T. S. Alster , “Complications of Cutaneous Laser Surgery. A Review,” Dermatologic Surgery 24, no. 2 (1998): 209–219. 9491115 10.1111/j.1524-4725.1998.tb04139.x

[78] M. A. Trelles , L. Pardo , O. Trelles , et al., “Clinical and Histologic Effects of Facial Skin Rejuvenation With Pulsed‐ and Continuous‐Wave Flash‐Scanned CO(2) Lasers,” Aesthetic Surgery Journal 21, no. 5 (2001): 399–411. 19331921 10.1067/maj.2001.119150

[79] N. Agrawal , G. Smith , and R. Heffelfinger , “Ablative Skin Resurfacing,” Facial Plastic Surgery 30, no. 1 (2014): 55–61. 24488638 10.1055/s-0033-1364223

[80] F. Seirafianpour , A. Pour Mohammad , Y. Moradi , et al., “Systematic Review and Meta‐Analysis of Randomized Clinical Trials Comparing Efficacy, Safety, and Satisfaction Between Ablative and Non‐Ablative Lasers in Facial and Hand Rejuvenation/Resurfacing,” Lasers in Medical Science 37, no. 4 (2022): 2111–2122. 35107665 10.1007/s10103-022-03516-0

[81] G. Palaia , L. D'Alessandro , D. Pergolini , R. Carletti , C. Di Gioia , and U. Romeo , “In Vivo Clinical and Histological Thermal Effect of a 445 nm Diode Laser on Oral Soft Tissues During a Biopsy,” Journal of Oral Science 63, no. 3 (2021): 280–282. 33980770 10.2334/josnusd.20-0665

[82] I. Kuzmina , I. Oshina , L. Dambite , et al., “Skin Chromophore Mapping by Smartphone RGB Camera Under Spectral Band and Spectral Line Illumination,” Journal of Biomedical Optics 27, no. 2 (2022): 026004. 35191236 10.1117/1.JBO.27.2.026004 PMC8860175

[83] S. Guida , C. Longo , S. Amato , et al., “Laser Treatment Monitoring With Reflectance Confocal Microscopy,” Medicina (Kaunas, Lithuania) 59, no. 6 (2023): 1039. 37374244 10.3390/medicina59061039 PMC10301319

[84] F. Medved , A. Wurm , and M. Held , “Facial Microcirculatory and Biomechanical Skin Properties After Single High Energy (Er):YAG Laser Application,” Lasers in Surgery and Medicine 49 (2017): 891–898, 10.1002/lsm.22710. 28799650

[85] L. Izikson , J. S. Nelson , and R. R. Anderson , “Treatment of Hypertrophic and Resistant Port Wine Stains With a 755 nm Laser: A Case Series of 20 Patients,” Lasers in Surgery and Medicine 41 (2009): 427–432, 10.1002/lsm.20793. 19588532 PMC2775078

[86] M. A. Alshami , “New Application of the Long‐Pulsed Nd‐YAG Laser as an Ablative Resurfacing Tool for Skin Rejuvenation: A 7‐Year Study,” Journal of Cosmetic Dermatology 12 (2013): 170–178, 10.1111/jocd.12052. 23992158

[87] L. Hernandez , N. Mohsin , F. S. Frech , I. Dreyfuss , A. Vander Does , and K. Nouri , “Laser Tattoo Removal: Laser Principles and an Updated Guide for Clinicians,” Lasers in Medical Science 37 (2022): 2581–2587, 10.1007/s10103-022-03576-2. 35604505

[88] A. A. Meesters , L. H. Pitassi , V. Campos , A. Wolkerstorfer , and C. C. Dierickx , “Transcutaneous Laser Treatment of Leg Veins,” Lasers in Medical Science 29, no. 2 (2014): 481–492. 24220848 10.1007/s10103-013-1483-2

[89] S. Karsai , S. Roos , S. Hammes , and C. Raulin , “Pulsed Dye Laser: What's New in Non‐Vascular Lesions,” Journal of the European Academy of Dermatology and Venereology 21, no. 7 (2007): 877–890. 17658995 10.1111/j.1468-3083.2007.02297.x

[90] D. Manstein , G. S. Herron , R. K. Sink , H. Tanner , and R. R. Anderson , “Fractional Photothermolysis: A New Concept for Cutaneous Remodeling Using Microscopic Patterns of Thermal Injury,” Lasers in Surgery and Medicine 34, no. 5 (2004): 426–438. 15216537 10.1002/lsm.20048

[91] I. Bogdan Allemann and J. Kaufman , “Fractional Photothermolysis,” Current Problems in Dermatology 42 (2011): 56–66. 21865799 10.1159/000328252

[92] B. Balaraman , P. Ravanfar‐Jordan , and P. M. Friedman , “Novel Use of Non‐Ablative Fractional Photothermolysis for Café‐Au‐Lait Macules in Darker Skin Types,” Lasers in Surgery and Medicine 49, no. 1 (2017): 84–87. 27388906 10.1002/lsm.22535

[93] M. G. Kharchilava , G. N. Ponomarenko , V. N. Plakhov , et al., “Experience With Fractional Photothermolysis in the Therapy of Gra‐Nuloma Annulare,” Voprosy Kurortologii, Fizioterapii, i Lechebnoĭ Fizicheskoĭ Kultury 96, no. 3 (2019): 60–63. 31329190 10.17116/kurort20199603160

[94] P. M. Friedman , K. D. Polder , P. Sodha , and R. G. Geronemus , “The 1440 nm and 1927 nm Nonablative Fractional Diode Laser: Current Trends and Future Directions,” Journal of Drugs in Dermatology 19, no. 8 (2020): s3–s11. 32804450

[95] B. Zou , W. Zheng , H. Pan , B. Yang , and Z. Liu , “Research Trends and Hotspot Analysis of Fractional Carbon Dioxide Laser: A Bibliometric and Visualized Analysis via Citespace,” Journal of Cosmetic Dermatology 21, no. 11 (2022): 5484–5499. 35869829 10.1111/jocd.15267

[96] K. E. Kim , J. Y. Jeong , J. Y. Jo , H. J. Ryu , and I. H. Kim , “Efficacy of Skin Rejuvenation With a Fractional 1927‐nm Thulium Laser Alone or Combined With a Chemical Peel: A Controlled Histopathological Preliminary Study in a Mouse Model,” Lasers in Medical Science 38, no. 1 (2023): 262. 37947906 10.1007/s10103-023-03928-6

[97] R. C. Patriota , C. J. Rodrigues , and L. C. Cucé , “Intense Pulsed Light in Photoaging: A Clinical, Histopathological and Immunohistochemical Evaluation,” Anais Brasileiros de Dermatologia 86, no. 6 (2011): 1129–1133. 22281900 10.1590/s0365-05962011000600010

[98] B. E. DiBernardo and J. N. Pozner , “Intense Pulsed Light Therapy for Skin Rejuvenation,” Clinics in Plastic Surgery 43, no. 3 (2016): 535–540. 27363767 10.1016/j.cps.2016.03.008

[99] C. Ping , D. Xueliang , L. Yongxuan , et al., “A Retrospective Study on the Clinical Efficacy of the Intense Pulsed Light Source for Photodamage and Skin Rejuvenation,” Journal of Cosmetic and Laser Therapy 18, no. 4 (2016): 217–224. 26734811 10.3109/14764172.2015.1114649

[100] A. Augustyniak and H. Rotsztejn , “Intense Pulsed Light (IPL) Treatment for the Skin in the Eye Area—Clinical and Cutometric Analysis,” Journal of Cosmetic and Laser Therapy 19, no. 1 (2017): 18–24. 27762643 10.1080/14764172.2016.1247963

[101] L. L. Faucz , S. E. Will , C. J. Rodrigues , H. Hesse , A. C. Moraes , and D. A. Maria , “Quantitative Evaluation of Collagen and Elastic Fibers After Intense Pulsed Light Treatment of Mouse Skin,” Lasers in Surgery and Medicine 50 (2018): 644–650. 10.1002/lsm.22782 29336034

[102] B. Barikbin , Z. Akbari , R. Vafaee , and Z. Razzaghi , “The Efficacy of IPL in Periorbital Skin Rejuvenation: An Open‐Label Study,” Journal of Lasers in Medical Sciences 10, no. Suppl 1 (2019): S64–S67. 32021676 10.15171/jlms.2019.S12 PMC6983864

[103] G. Cannarozzo , D. Bonciani , F. Tamburi , et al., “New Insight in Noninvasive Rejuvenation: The Role of a Rhodamine‐Intense Pulsed Light System,” Photobiomodulation, Photomedicine, and Laser Surgery 37, no. 9 (2019): 539–543. 31381488 10.1089/photob.2019.4626

[104] J. M. Knight and G. Kautz , “Sequential Facial Skin Rejuvenation With Intense Pulsed Light and Non‐Ablative Fractionated Laser Resurfacing in Fitzpatrick Skin Type II–IV Patients: A Prospective Multicenter Analysis,” Lasers in Surgery and Medicine 51, no. 2 (2019): 141–149. 30091207 10.1002/lsm.23007 PMC6585794

[105] J. Kim , J. Lee , and H. Choi , “Intense Pulsed Light Attenuates UV‐Induced Hyperimmune Response and Pigmentation in Human Skin Cells,” International Journal of Molecular Sciences 22, no. 6 (2021): 3173. 33804685 10.3390/ijms22063173 PMC8003787

[106] A. Sales , I. L. Pandolfo , C. M. de Almeida , et al., “Intense Pulsed Light on Skin Rejuvenation: A Systematic Review,” Archives of Dermatological Research 314, no. 9 (2022): 823–838. 34609598 10.1007/s00403-021-02283-2

[107] R. Zein , W. Selting , and M. R. Hamblin , “Review of Light Parameters and Photobiomodulation Efficacy: Dive Into Complexity,” Journal of Biomedical Optics 23, no. 12 (2018): 1–17. 10.1117/1.JBO.23.12.120901 PMC8355782 30550048

[108] L. Kirsanova , E. Araviiskaia , M. Rybakova , E. Sokolovsky , A. Bogantenkov , and F. Al‐Niaimi , “Histological Characterization of Age‐Related Skin Changes Following the Use of Picosecond Laser: Low vs High Energy,” Dermatologic Therapy 33, no. 4 (2020): e13635. 32436343 10.1111/dth.13635

[109] C. Nanni , “Complications of Laser Surgery,” Dermatologic Clinics 15, no. 3 (1997): 521–534. 9189687 10.1016/s0733-8635(05)70459-9

[110] S. Watanabe , “Basics of Laser Application to Dermatology,” Archives of Dermatological Research 300, no. Suppl 1 (2008): S21–S30. 17962966 10.1007/s00403-007-0801-6

[111] Z. F. Jasim and J. M. Handley , “Treatment of Pulsed Dye Laser‐Resistant Port Wine Stain Birthmarks,” Journal of the American Academy of Dermatology 57 (2007): 677–682, 10.1016/j.jaad.2007.01.019. 17658196

[112] H. C. Jo and D. Y. Kim , “Observations of In Vivo Laser Tissue Ablation in Animal Models With Different Chromophores on the Skin and Modulating Duration per Laser Exposure,” Lasers in Medical Science 34, no. 5 (2019): 1031–1039. 30488272 10.1007/s10103-018-2693-4

[113] E. Victor Ross , H. Chodkiewicz , S. Javvaji , J. Zumwalt , T. D. Kutscher , and C. Tran , “Enhanced Pulsed Dye Laser for Facial Rejuvenation,” Lasers in Surgery and Medicine 53 (2021): 109–114, 10.1002/lsm.23309. 32779273

[114] Y. Cai , X. Zeng , J. Ying , Y. Zhu , Y. Qiu , and W. Xiang , “Efficacy and Safety of Pulsed Dye Laser for the Treatment of Surgical Scars: A Systematic Review and Meta‐Analysis,” Lasers in Medical Science 37 (2022): 1273–1282, 10.1007/s10103-021-03385-z. 34351564

[115] J. S. Nelson , B. Majaron , and K. M. Kelly , “Active Skin Cooling in Conjunction With Laser Dermatologic Surgery,” Seminars in Cutaneous Medicine and Surgery 19, no. 4 (2000): 253–266. 11149606 10.1053/sder.2000.18365

[116] A. Das , A. Sarda , and A. De , “Cooling Devices in Laser Therapy,” Journal of Cutaneous and Aesthetic Surgery 9, no. 4 (2016): 215–219. 28163450 10.4103/0974-2077.197028 PMC5227072

[117] E. Russe , M. Purschke , M. Herold , F. H. Sakamoto , G. Wechselberger , and K. Russe‐Wilflingseder , “Evaluation of Safety and Efficacy of Laser Hair Removal With the Long‐Pulsed 755 Nm Wavelength Laser: A Two‐Center Study With 948 Patients,” Lasers in Surgery and Medicine 52, no. 1 (2020): 77–83. 31579971 10.1002/lsm.23160

[118] H. Kim , J. K. Hwang , J. Choi , and H. W. Kang , “Dependence of Laser‐Induced Optical Breakdown on Skin Type During 1064 Nm Picosecond Laser Treatment,” Journal of Biophotonics 14 (2021): e202100129, 10.1002/jbio.202100129. 34114344

[119] P. H. Wardhani , C. Prakoeswa , and M. Y. Listiawan , “Efficacy and Safety of Picosecond Laser for Wrinkle in Indonesian Skin,” Journal of Cosmetic and Laser Therapy 24 (2022): 33–35, 10.1080/14764172.2022.2074038. 35603678

[120] P. H. Wardhani , C. R. Sigit Prakoeswa , and M. Y. Listiawan , “Facial Rejuvenation in Indonesian Skin With a Picosecond 755‐nm Laser,” Journal of Lasers in Medical Sciences 13 (2022): e45, 10.34172/jlms.2022.45. 36743150 PMC9841370

[121] J. M. Amici , O. Cogrel , M. Jourdan , et al., “Expert Recommendations on Supportive Skin Care for Non‐Surgical and Surgical Procedures,” Journal of the European Academy of Dermatology and Venereology 37, no. Suppl 3 (2023): 16–33, 10.1111/jdv.18855. 36635618

[122] J. R. Chao , J. P. Porter , and J. Hessler , “Cosmetic Treatments With Energy‐Based Devices in Skin of Color,” Facial Plastic Surgery 39 (2023): 496–500, 10.1055/s-0043-1772197. 37557909

[123] F. J. Eckembrecher , D. G. Eckembrecher , I. Camacho , H. Shah , D. Jaalouk , and K. Nouri , “A Review of Treatment of Port‐Wine Stains With Pulsed Dye Laser in Fitzpatrick Skin Type IV–VI,” Archives of Dermatological Research 315 (2023): 2505–2511, 10.1007/s00403-023-02640-3. 37253863

[124] M. H. Gold , E. Weiss , and J. Biron , “Novel Laser Hair Removal in All Skin Types,” Journal of Cosmetic Dermatology 22 (2023): 1261–1265, 10.1111/jocd.15674. 36756716

[125] M. Sowash and T. Alster , “Review of Laser Treatments for Post‐Inflammatory Hyperpigmentation in Skin of Color,” American Journal of Clinical Dermatology 24 (2023): 381–396, 10.1007/s40257-023-00759-7. 36781686

[126] N. S. Sadick and Y. Makino , “Selective Electro‐Thermolysis in Aesthetic Medicine: A Review,” Lasers in Surgery and Medicine 34 (2004): 91–97, 10.1002/lsm.20013. 15004818

[127] D. V. Wakade , C. S. Nayak , and K. D. Bhatt , “A Study Comparing the Efficacy of Monopolar Radiofrequency and Glycolic Acid Peels in Facial Rejuvenation of Aging Skin Using Histopathology and Ultrabiomicroscopic Sonography (UBM)—An Evidence Based Study,” Acta Medica (Hradec Králové) 59, no. 1 (2016): 14–17. 27131351 10.14712/18059694.2016.49

[128] A. R. Bonjorno , T. B. Gomes , M. C. Pereira , et al., “Radiofrequency Therapy in Esthetic Dermatology: A Review of Clinical Evidences,” Journal of Cosmetic Dermatology 19, no. 2 (2020): 278–281. 31691477 10.1111/jocd.13206

[129] M. H. Gold , J. Biron , and A. Wilson , “Improvement of Skin Texture and Wrinkles Using Radiofrequency Ultra‐Thin Electrode Technology,” Journal of Cosmetic Dermatology 19, no. 2 (2020): 388–392. 31829510 10.1111/jocd.13239

[130] S. Bhargava , M. Goldust , H. Singer , N. Negbenebor , and G. Kroumpouzos , “Evaluating Resurfacing Modalities in Aesthetics,” Clinics in Dermatology 40, no. 3 (2022): 274–282. 35667824 10.1016/j.clindermatol.2021.01.019

[131] A. S. Stochaj , D. H. Jezierska , and L. Kubisz , “Comparing the Efficacy of Monopolar and Bipolar Radiofrequency Treatment on Facial Skin in Women,” Journal of Clinical and Aesthetic Dermatology 15, no. 12 (2022): 22–27. PMC9762629 36569525

[132] S. Garg , K. R. Vashisht , P. R. Sushruth , and D. V. Saka , “Monopolar Radiofrequency for Reposing Drooping Fat Planes and Facial Rejuvenation: A Prospective Study on 30 Subjects Towards True Antiaging,” Journal of Cosmetic Dermatology 21 (2022): 1489–1500, 10.1111/jocd.14255. 34038038

[133] M. S. Lolis and D. J. Goldberg , “Radiofrequency in Cosmetic Dermatology: A Review,” Dermatologic Surgery 38, no. 11 (2012): 1765–1776. 22913399 10.1111/j.1524-4725.2012.02547.x

[134] O. A. Ogbechie‐Godec and N. Elbuluk , “Melasma: An Up‐To‐Date Comprehensive Review,” Dermatology and Therapy 7, no. 3 (2017): 305–318. 28726212 10.1007/s13555-017-0194-1 PMC5574745

[135] S. Bhargava , P. R. Cunha , J. Lee , and G. Kroumpouzos , “Acne Scarring Management: Systematic Review and Evaluation of the Evidence,” American Journal of Clinical Dermatology 19, no. 4 (2018): 459–477. 29744784 10.1007/s40257-018-0358-5

[136] J. M. Garden , B. Zelickson , D. Friedman , T. D. Kutscher , D. M. Rozen , and V. Afsahi , “Long‐Term Facial and Body Hair Removal With a Combined Radiofrequency and Optical Home‐Use Device for All Skin Types,” Journal of Drugs in Dermatology 19, no. 5 (2020): 498–503. 32484632

[137] M. G. Tan , C. E. Jo , A. Chapas , S. Khetarpal , and J. S. Dover , “Radiofrequency Microneedling: A Comprehensive and Critical Review,” Dermatologic Surgery: Official Publication for American Society for Dermatologic 47 (2021): 755–761, 10.1097/DSS.0000000000002972. 33577211

[138] M. Cohen , E. Austin , N. Masub , A. Kurtti , C. George , and J. Jagdeo , “Home‐Based Devices in Dermatology: A Systematic Review of Safety and Efficacy,” Archives of Dermatological Research 314, no. 3 (2022): 239–246. 33938981 10.1007/s00403-021-02231-0 PMC8918178

[139] H. M. Kim , S. Oh , K. A. Byun , et al., “Radiofrequency Irradiation Mitigated UV‐B‐Induced Skin Pigmentation by Increasing Lymphangiogenesis,” Molecules 27, no. 2 (2022): 454. 35056769 10.3390/molecules27020454 PMC8780734

[140] K. A. Byun , H. M. Kim , S. Oh , K. H. Son , and K. Byun , “Radiofrequency Irradiation Attenuated UVB‐Induced Skin Pigmentation by Modulating ATP Release and CD39 Expression,” International Journal of Molecular Sciences 24, no. 6 (2023): 5506. 36982581 10.3390/ijms24065506 PMC10052073

[141] D. Berube , B. Renton , and B. M. Hantash , “A Predictive Model of Minimally Invasive Bipolar Fractional Radiofrequency Skin Treatment,” Lasers in Surgery and Medicine 41, no. 7 (2009): 473–478. 19708063 10.1002/lsm.20794

[142] D. Mazzoni , M. J. Lin , D. P. Dubin , and H. Khorasani , “Review of non‐invasive body contouring devices for fat reduction, skin tightening and muscle definition,” Australasian Journal of Dermatology 60, no. 4 (2019): 278–283, 10.1111/ajd.13090. 31168833

[143] M. Kiedrowicz , E. Duchnik , J. Wesołowska , et al., “Early and Long‐Term Effects of Abdominal Fat Reduction Using Ultrasound and Radiofrequency Treatments,” Nutrients 14 (2022): 3498, 10.3390/nu14173498. 36079758 PMC9459719

[144] J. Wolska and H. Hassan , “Noninvasive Lipolysis Modalities in Aesthetic Medicine,” Journal of Cosmetic Dermatology 22 (2023): 2635–2649, 10.1111/jocd.15929. 37431699

[145] E. Dayan , C. Chia , A. J. Burns , and S. Theodorou , “Adjustable Depth Fractional Radiofrequency Combined With Bipolar Radiofrequency: A Minimally Invasive Combination Treatment for Skin Laxity,” Aesthetic Surgery Journal 39, no. Suppl_3 (2019): S112–S119. 30958550 10.1093/asj/sjz055 PMC6460431

[146] S. F. Weiner , “Radiofrequency Microneedling: Overview of Technology, Advantages, Differences in Devices, Studies, and Indications,” Facial Plastic Surgery Clinics of North America 27, no. 3 (2019): 291–303. 31280844 10.1016/j.fsc.2019.03.002

[147] D. Alessa and J. D. Bloom , “Microneedling Options for Skin Rejuvenation, Including Non‐Temperature‐Controlled Fractional Microneedle Radiofrequency Treatments,” Facial Plastic Surgery Clinics of North America 28, no. 1 (2020): 1–7. 31779933 10.1016/j.fsc.2019.09.001

[148] A. J. Hendricks and S. Z. Farhang , “Dermatologic Facial Applications of Morpheus8 Fractional Radiofrequency Microneedling,” Journal of Cosmetic Dermatology 21, no. Suppl 1 (2022): S11–S19. 35916259 10.1111/jocd.15231

[149] E. A. Spataro , K. Dierks , and P. J. Carniol , “Microneedling‐Associated Procedures to Enhance Facial Rejuvenation,” Facial Plastic Surgery Clinics of North America 30, no. 3 (2022): 389–397. 35934440 10.1016/j.fsc.2022.03.012

[150] A. Sturm , T. Shokri , and Y. Ducic , “Nonsurgical Rejuvenation of the Neck,” Clinics in Plastic Surgery 50, no. 3 (2023): 497–507. 37169415 10.1016/j.cps.2022.12.014

[151] Z. Alizadeh , F. Halabchi , R. Mazaheri , M. Abolhasani , and M. Tabesh , “Review of the Mechanisms and Effects of Noninvasive Body Contouring Devices on Cellulite and Subcutaneous Fat,” International Journal of Endocrinology and Metabolism 14 (2016): e36727, 10.5812/ijem.36727. 28123436 PMC5236497

[152] W. M. White , I. R. Makin , P. G. Barthe , M. H. Slayton , and R. E. Gliklich , “Selective Creation of Thermal Injury Zones in the Superficial Musculoaponeurotic System Using Intense Ultrasound Therapy: A New Target for Noninvasive Facial Rejuvenation,” Archives of Facial Plastic Surgery 9 (2007): 22–29, 10.1001/archfaci.9.1.22. 17224484

[153] M. Kerscher , A. T. Nurrisyanti , C. Eiben‐Nielson , S. Hartmann , and J. Lambert‐Baumann , “Skin Physiology and Safety of Microfocused Ultrasound With Visualization for Improving Skin Laxity,” Clinical, Cosmetic and Investigational Dermatology 12 (2019): 71–79, 10.2147/CCID.S188586. 30666145 PMC6336023

[154] U. Khan and N. Khalid , “A Systematic Review of the Clinical Efficacy of Micro‐Focused Ultrasound Treatment for Skin Rejuvenation and Tightening,” Cureus 13 (2021): e20163, 10.7759/cureus.20163. 35003992 PMC8722640

[155] M. Contini , M. Hollander , A. Vissink , R. H. Schepers , J. Jansma , and J. Schortinghuis , “A Systematic Review of the Efficacy of Microfocused Ultrasound for Facial Skin Tightening,” International Journal of Environmental Research and Public Health 20 (2023): 1522, 10.3390/ijerph20021522. 36674277 PMC9861614

[156] Z. Kyriakou , M. I. Corral‐Baques , A. Amat , and C. C. Coussios , “HIFU‐Induced Cavitation and Heating in Ex Vivo Porcine Subcutaneous Fat,” Ultrasound in Medicine & Biology 37 (2011): 568–579, 10.1016/j.ultrasmedbio.2011.01.001. 21371810

[157] Z. Alizadeh , F. Halabchi , Z. Bodaghabadi , et al., “Non‐Invasive Body Contouring Technologies: An Updated Narrative Review,” Aesthetic Plastic Surgery 48, no. 4 (2024): 659–679. 37749418 10.1007/s00266-023-03647-x

[158] S. Oh , D. Y. Rhee , S. Batsukh , K. H. Son , and K. Byun , “High‐Intensity Focused Ultrasound Increases Collagen and Elastin Fiber Synthesis by Modulating Caveolin‐1 in Aging Skin,” Cells 12, no. 18 (2023): 2275. 37759497 10.3390/cells12182275 PMC10527789

[159] B. Katz , “Concomitant Use of Radiofrequency and High Intensity Focused Electromagnetic Field Energies for Full‐Body Remodeling: MRI Evidence‐Based Prefatory Trial,” Journal of Cosmetic Dermatology 22, no. 1 (2023): 193–199. 36045514 10.1111/jocd.14736 PMC10087156

[160] D. E. Kent , C. Jacob , and B. M. Kinney , “Retrospective Analysis of High‐Intensity Focused Electromagnetic Procedure Synchronized With Radiofrequency Energy for Visceral Fat Reduction,” Journal of Cosmetic Dermatology 22, no. 9 (2023): 2485–2491. 37154787 10.1111/jocd.15784

[161] Y. Halaas and J. Bernardy , “Mechanism of Nonthermal Induction of Apoptosis by High‐Intensity Focused Electromagnetic Procedure: Biochemical Investigation in a Porcine Model,” Journal of Cosmetic Dermatology 19, no. 3 (2020): 605–611. 31943721 10.1111/jocd.13295 PMC7028149

[162] M. Tai , J. Chen , J. Chen , X. Shen , and J. Ni , “Endoplasmic Reticulum Stress in Skin Aging Induced by UVB,” Experimental Dermatology 33, no. 1 (2024): e14956. 37846942 10.1111/exd.14956

[163] J. Kohan , K. Vyas , M. Erotocritou , A. Khajuria , and K. Tehrani , “High‐Intensity Focused Electromagnetic (HIFEM) Energy With and Without Radiofrequency for Noninvasive Body Contouring: A Systematic Review,” Aesthetic Plastic Surgery 48, no. 6 (2024): 1156–1165. 37957393 10.1007/s00266-023-03730-3

[164] D. E. Kent and C. I. Jacob , “Simultaneous Changes in Abdominal Adipose and Muscle Tissues Following Treatments by High‐Intensity Focused Electromagnetic (HIFEM) Technology‐Based Device: Computed Tomography Evaluation,” Journal of Drugs in Dermatology 18, no. 11 (2019): 1098–1102. 31738500

[165] D. I. Duncan and M. Busso , “Effectiveness of Combined Use of Targeted Pressure Energy, Radiofrequency, and High‐Intensity Focused Electromagnetic Fields to Improve Skin Quality and Appearance of Fat and Muscle Tissue in Different Body Parts,” Journal of Cosmetic Dermatology 22, no. 1 (2023): 200–205. 36045512 10.1111/jocd.15280 PMC10087797

[166] J. B. Samuels , B. Katz , and R. A. Weiss , “Radiofrequency Heating and High‐Intensity Focused Electromagnetic Treatment Delivered Simultaneously: The First Sham‐Controlled Randomized Trial,” Plastic and Reconstructive Surgery 149, no. 5 (2022): 893e–900e. 10.1097/PRS.0000000000009030 PMC9028295 35259147

[167] B. M. Kinney , J. Bernardy , and R. Jarošová , “Novel Technology for Facial Muscle Stimulation Combined With Synchronized Radiofrequency Induces Structural Changes in Muscle Tissue: Porcine Histology Study,” Aesthetic Surgery Journal 43, no. 8 (2023): 920–927. 36883601 10.1093/asj/sjad053 PMC10712423

[168] S. B. Kim , S. Kim , Y. R. Heo , and H. J. Kim , “Evaluation of a Novel Device Combining RF and HIFES Technologies for the Non‐Invasive Correction of Asymmetric Smiles and Facial Rejuvenation: A Case Report,” Skin Research and Technology 30, no. 8 (2024): e13885. 39120930 10.1111/srt.13885 PMC11313414

[169] B. M. Kinney and C. M. Boyd , “Remodeling of Facial Soft Tissue Induced by Simultaneous Application of HIFES and Synchronized Radiofrequency Provides Nonsurgical Lift of Facial Soft Tissues,” Journal of Cosmetic Dermatology 23, no. 3 (2024): 824–829. 38235951 10.1111/jocd.16165

[170] O. A. Ibrahimi , K. J. Stankiewicz , H. R. Jalian , and S. S. Saluja , “High‐Intensity Focused Radio Frequency Is Safe and Effective for the Treatment of Acne Scars in Skin of Color,” Dermatologic Surgery 47, no. 6 (2021): 860–861. 33481444 10.1097/DSS.0000000000002848

[171] C. Haut , “New Technologies for Noninvasive Aesthetic Treatments,” Dermatologie 74, no. 10 (2023): 759–764. 37650892 10.1007/s00105-023-05205-7

